# Optimization of Clustering and Trajectory for Minimizing Age of Information in Unmanned Aerial Vehicle-Assisted Mobile Edge Computing Network

**DOI:** 10.3390/s24061742

**Published:** 2024-03-07

**Authors:** Huicong Shen, Die Wang, Zhen Huang, Yunjian Jia

**Affiliations:** School of Microelectronics and Communication Engineering, Chongqing University, Chongqing 401331, China; huicongshen@stu.cqu.edu.cn (H.S.); wangdie@cqu.edu.cn (D.W.); zhenhuang@cqu.edu.cn (Z.H.)

**Keywords:** Internet of Things, mobile edge computing, unmanned aerial vehicle, age of information, clustering, trajectory

## Abstract

With the development of the Internet of Things (IoT) technology, massive amounts of sensor data in applications such as fire monitoring need to be transmitted to edge servers for timely processing. However, there is an energy-hole phenomenon in transmitting data only through terrestrial multi-hop networks. In this study, we focus on the data collection task in an unmanned aerial vehicle (UAV)-assisted mobile edge computing (MEC) network, where a UAV is deployed as the mobile data collector for the ground sensor nodes (SNs) to ensure high information freshness. Meanwhile, the UAV is equipped with an edge server for data caching. We first establish a rigorous mathematical model in which the age of information (AoI) is used as a measure of information freshness, related to both the data collection time and the UAV’s flight time. Then a mixed-integer non-convex optimization problem is formulated to minimize the peak AoI of the collected data. To solve the problem efficiently, we propose an iterative two-step algorithm named the AoI-minimized association and trajectory planning (AoI-MATP) algorithm. In each iteration, the optimal SN–collection point (CP) associations and CP locations for the parameter ε are first obtained by the affinity propagation clustering algorithm. The optimal UAV trajectory is found using an improved elite genetic algorithm. Simulation results show that based on the optimized ε, the AoI-MATP algorithm can achieve a balance between data collection time and flight time, reducing the peak AoI of the collected data.

## 1. Introduction

In the era of 5G, the flourishing progress of the Internet of Things (IoT) technology is instigating transformative shifts in emerging application domains. Leveraging the high-speed and low-latency attributes of 5G networks [1], wireless sensor networks (WSNs) containing a large number of sensors have become the backbone of the IoT, demonstrating an impressive capability to transmit massive amounts of data with exceptional efficiency. Consequently, WSNs can provide unprecedented real-time sensing and decision support for status-update systems with stringent real-time demands. It has emerged as the central driving force behind applications such as smart transportation [2], environmental monitoring [3], and smart agriculture [4]. However, ground sensor nodes (SNs) used for monitoring in WSNs usually suffer from small size and limited battery capacity. In some remote areas, SNs are far away from data centers. Transmitting the data sensed by SNs only through the ground-based multi-hop network is prone to the energy hole phenomenon [5]. It cannot satisfy the high information freshness in the state update system, affecting the timeliness and accuracy of decision-making.

Therefore, in real-time sensing scenarios such as forest fire monitoring [6] and disaster monitoring [7], the use of mobile data collectors like unmanned aerial vehicles (UAV) is considered a promising solution [8]. Recently, UAVs have attracted extensive attention due to their significant advantages [9,10]. First, UAVs can fly to inaccessible places to collect data, improving the completeness and accuracy of data collection compared to traditional collectors. Second, they have a high flight speed and bandwidth, making data collection fast and efficient. Third, they can be equipped with mobile edge computing (MEC) servers for real-time sensing, data processing, and decision-making. By processing data instantly on the fly, the latency of data transmission to the cloud is reduced, resulting in a more rapid response. Moreover, since UAVs usually have limited computing, storage, and power resources, some computationally intensive tasks may exceed their processing capabilities. Equipping MEC servers can provide additional computational and storage resources to optimize the efficiency of task execution. Finally, they can move close to the ground SNs, making it easy to establish line of sight (LoS) links and reduce the transmit power and packet loss rate [11]. Currently, data collection in UAV-assisted MEC networks has become a popular research topic, and scholars have carried out a lot of work accordingly.

Due to the limited energy onboard the UAV, a part of the existing work focuses on minimizing energy consumption in data collection tasks [12,13,14,15,16]. Some scholars have considered the impact of different data collection modes on UAV energy consumption, such as hover mode [12], flight mode [13], and hybrid mode [14]. In comparison, the flight mode has the lowest energy consumption but requires a long task completion time, while the hover mode requires higher energy consumption but achieves the minimum task completion time. In [15], a framework for data collection in UAV-assisted WSNs was considered and a sensor-energy-based method for initial UAV trajectory determination was proposed to maximize the minimum residual energy of the SNs after data transmission. To improve the security and energy efficiency of data collection in IoT applications, an adaptive linear prediction algorithm was designed to reduce the energy consumption of the WSN [16]. Furthermore, in [17], a binary search algorithm based on successive convex approximation (SCA) was designed to minimize the collection time, and the Frequency Division Multiple Access (FDMA) scheme was found to be superior to the simpler Time Division Multiple Access (TDMA) scheme by comparison. The algorithm proposed in [18] maximized the minimum data collection rate by jointly optimizing the 3D trajectory of the UAV and the time allocation.

However, in state update systems, outdated data can lead to wrong decisions, so there is an extremely high requirement for data freshness. Generally, the age of information (AoI) is used to characterize data freshness, which portrays the time elapsed since the generation of the latest incoming update [19]. Therefore, many scholars have considered minimizing the peak AoI or average AoI in data collection tasks. On the one hand, when the number of sensors is large, the data collection strategy plays an important role in reducing the AoI. References [20,21,22,23,24] utilized clustering algorithms to divide the network into multiple sub-networks, and the UAV only needed to collect data at the corresponding data collection points (CPs) of each sub-network, thus reducing the AoI. Commonly used algorithms include simple adaptive algorithms [22], k-means clustering algorithms [23], and affinity propagation (AP) algorithms [24]. For different system models and performance metrics, it is necessary to choose the appropriate clustering algorithm. Moreover, the authors proposed an aerial collaborative relay-based data collection scheme to obtain better AoI performance in [25]. In [26], the data collection method, the energy consumption, and the age evolution were comprehensively optimized to minimize the average AoI.

On the other hand, the UAV’s flight trajectory greatly affects the time required for the data collection task. The high flexibility of UAVs imposes challenges on their trajectory planning, and much attention has been paid to deriving the optimal trajectory of UAVs from source to destination under given conditions. The authors of [27] jointly optimized the UAV’s trajectory and the service time allocation to minimize the SN’s average AoI. In [28], an algorithm based on graph theory and the kernel K-means method and an energy-constrained trajectory planning algorithm were proposed to reduce the average AoI and the energy consumption of each UAV. The authors jointly optimized the collection time and the trajectory of the UAV to achieve a trade-off between AoI and energy consumption in [29]. By decomposing the optimization problem, the sub-problems were solved using SCA and Lagrange duality methods, respectively. An iterative algorithm was proposed for minimizing the peak and average AoI of multi-UAV-assisted data collection in [30], where an improved ant colony optimization (ACO) algorithm was used to optimize the UAVs’ trajectories. In [31], the authors proposed an average AoI-minimizing trajectory design based on the Safe-Twin Delayed Deep Deterministic policy gradient. In general, UAV trajectory planning algorithms can be broadly classified into graph theory-based algorithms, optimization theory-based algorithms, and artificial intelligence (AI)-based algorithms in UAV-assisted data collection scenarios. Since the computational complexity of traditional optimization-based algorithms mostly grows exponentially with the system size, heuristic algorithms such as the ACO algorithm and genetic algorithm (GA) become better choices that can obtain a near-optimal solution in a shorter time. However, they are not suitable for real-time planning in rapidly changing environments, making online trajectory optimization considered to be implemented using machine learning algorithms [32].

The findings of the above work show that the collection method and the trajectory of the UAV have a significant impact on the AoI performance in data collection tasks. However, in related work [25,27,33], the non-line of sight (NLoS) communication link between the UAV and the ground SN was ignored, resulting in a model that may not be universal. Our work, on the other hand, builds a more rigorous mathematical model based on actual scenarios by taking into account the probability of both LoS and NLoS links occurring. Moreover, considering the case of a large number of SNs, different from directly performing trajectory planning [34], we first adopt a suitable clustering algorithm to divide the SNs into clusters. Meanwhile, similar work [24] failed to strike a balance between the accuracy and computational complexity of the solution, resulting in the computational complexity becoming intolerable when the problem size is large. Accordingly, our work is inspired to combine SN clustering with heuristic trajectory planning algorithms. The optimal collection strategies and offline flight trajectories for data collection scenarios in UAV-assisted MEC networks are investigated to minimize the peak AoI of the collected data, and the main contributions are as follows:

- We establish a rigorous mathematical model for the considered UAV-assisted MEC network, and we formulate a minimization problem for the peak AoI of the collected data, which is solved by jointly optimizing the SN–collection point (CP) association, the positions of the CPs, and the UAV trajectory.
- To solve this non-convex optimization problem accurately and efficiently, we propose a two-step iterative solution algorithm. First, an AP-based algorithm is applied to solve the optimal SN-CP associations and locations of CPs. In the second step, an improved elite genetic algorithm is used to obtain the AoI-optimal UAV trajectory.
- We control the number of clusters by varying the clustering parameter ε to achieve an optimal balance between data collection time and flight time. Accordingly, the optimal solution can be obtained by the ε that minimizes the peak AoI.

**Figure 1:**
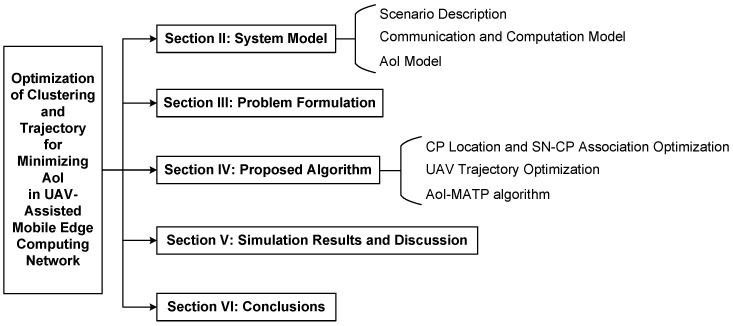
The overall framework diagram of this article.

The remainder of this article is organized as follows. In Section 2, the system model for UAV-assisted data collection in MEC networks is presented. Section 3 formulates a minimization problem on the peak AoI of SNs’ collected data. Section 4 describes the proposed iterative optimization algorithms in detail. Section 5 demonstrates the simulation results to evaluate the performance of the proposed algorithms. Finally, this article is concluded in Section 6. The overall framework diagram of this article is presented in Figure 1.

Notations: In this paper, scalars are denoted by italic letters and sets by calligraphic letters, such as M. Furthermore, vectors and matrices are denoted by bold letters. $R^{M\times K}$ denotes the space of M×K-dimensional real-valued matrices. $∥\cdot ∥$ denotes the Euclidean norm of a vector. |·| represents the cardinality of a set, i.e., the number of elements in a finite set. [·] denotes an array, i.e., an ordered sequence of elements. O(·) denotes the standard big-*O* notation.

## 2. System Model

### 2.1. Scenario Description

**Figure 2:**
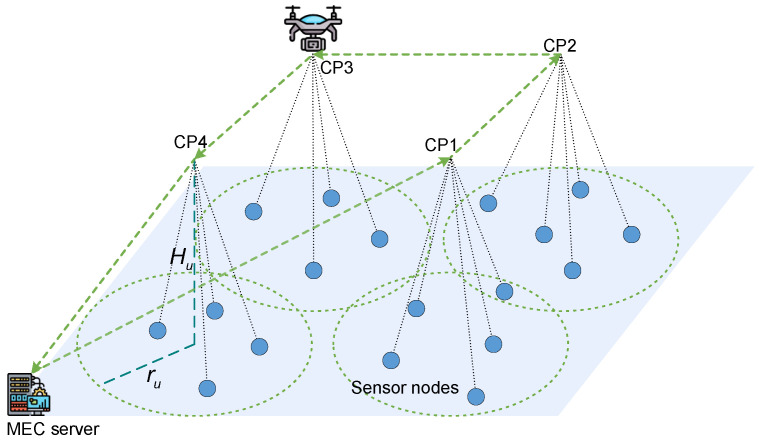
Illustration of the system scenario when K=4.

We consider a UAV-assisted MEC network for fire monitoring, as shown in Figure 2, in which a rotary-wing UAV is deployed as a mobile data collector for the *M* ground SNs. The UAV is outfitted with an edge server to enable real-time environmental sensing and data caching. It is assumed that the UAV has ample storage for the collected data, which it then transmits to the MEC server $s_{0}$, thereby facilitating subsequent information processing steps. The SNs are denoted by M={1,2,⋯,M}. Within a two-dimensional Cartesian coordinate system, $s_{0}$ occupies a fixed position at $w_{0}=\left(0,0\right)$. The SNs are scattered randomly across the specified geographical area, and the location of SN *m* is expressed as $w_{m}=\left(x_{m},y_{m}\right)$. The UAV maintains a constant flight altitude $H_{u}$, ensuring operational safety by avoiding collisions with other environmental objects. Data collection by the UAV is conducted across a set of data CPs, denoted by K={1,2,⋯,K}, where *K* is an element in the set M, i.e., K∈M. The coordinates of CP *k* projected on the ground are denoted by $u_{k}=\left(X_{k},Y_{k}\right)$.

Note that during the data collection phase, the SNs are grouped into *K* clusters corresponding to the CPs. The UAV starts from $s_{0}$ and sequentially visits *K* CPs to collect data, subsequently returning to $s_{0}$ to conclude a data collection cycle. Specifically, at each CP, the UAV hovers to establish communication links with all detectable SNs within that vicinity until all pertinent data are captured. Then the UAV flies at a constant speed *V* to the next CP. The monitoring radius accessible to the UAV is denoted by $r_{u}$. To ensure accurate data collection, the distance between CP *k* and any associated SN *m* should satisfy the following conditions:

(1) $$\begin{array}{c} ∥w_{m}-u_{k}∥\leq r_{u},\;m\in M,\;k\in K. \end{array}$$

For ease of exposition, the binary indicator $o_{m}^{k}$ is used to represent the association relationship between any CP *k* and SN *m*, i.e., whether the data of SN *m* can be collected when the UAV hovers at CP *k*. More specifically, $o_{m}^{k}=1$ when SN *m* is associated with CP *k*; otherwise, $o_{m}^{k}=0$. Then, the association relationship between the SNs and CPs can be denoted by a sparse matrix $o\in R^{M\times K}$. At each CP, the UAV can cover multiple SNs, but each SN should only be associated with one CP. Therefore, for any SN *m*, we have

(2) $$\begin{array}{c} \sum_{k=1}^{K}o_{m}^{k}=1,\;k\in K,\;m\in M, \end{array}$$

(3) $$\begin{array}{c} o_{m}^{k}\in \left\{0,1\right\},\;k\in K,\;m\in M. \end{array}$$

Let $A_{k}$ indicate the set of SNs associated with CP *k*; then, the expression $A_{k}=\left\{m\;|\;o_{m}^{k}=1,\;m\in M\right\}$ holds. The cardinality of the set $A_{k}$ is denoted by $|A_{k}|$, which in this case parallels the number of SNs associated with CP *k*.

### 2.2. Communication and Computation Model

In this section, a popular ground-to-air path loss model is used to illustrate the communication between the UAV and SNs on the ground. Specifically, the distance between CP *k* and SN *m* is denoted $d_{m}^{k}$, which can be obtained by $d_{m}^{k}=\sqrt{{∥w_{m}-u_{k}∥}^{2}+{H_{u}}^{2}}$. Our model takes into account both LoS and NLoS links between the UAV and its target. It should be noted that LoS links and NLoS links are two fundamental types of signal propagation paths in wireless communication. LoS links refer to situations where there is a direct line of sight between the transmitter and receiver, allowing wireless signals to propagate directly without any obstructions. These links typically offer higher signal quality and lower propagation loss. NLoS links refer to situations where there is no direct line of sight between the transmitter and receiver, requiring signals to reach the receiver through diffraction, reflection, or refraction. These links may encounter more attenuation and interference during signal propagation. Referring to the widely used channel model in [35,36], the probability of an LoS link occurring can be obtained by $P_{LoS}\left(d_{m}^{k}\right)=\frac{1}{1+a\;\exp(-b\left(\theta \left(d_{m}^{k}\right)-a\right))}$, where *a* and *b* are constant parameters determined by the environment and $\theta (d_{m}^{k})$ is the degree of elevation angle between the UAV and the SN, expressed as $\theta \left(d_{m}^{k}\right)=\frac{180}{\pi }\arcsin\left(\frac{H_{u}}{d_{m}^{k}}\right)$. The probability of an NLoS link occurring can be given by $P_{NLoS}\left(d_{m}^{k}\right)=1-P_{LoS}\left(d_{m}^{k}\right)$.

Meanwhile, the channel coefficient between SN *m* and the UAV hovering at CP *k* is denoted $h(d_{m}^{k})$. It can be derived from $h\left(d_{m}^{k}\right)=\sqrt{\beta (d_{m}^{k})}\lambda \left(d_{m}^{k}\right)$, where $\beta (d_{m}^{k})$ reflects the large-scale propagation effects of the channel, such as path loss and shadowing, and $\lambda (d_{m}^{k})$ reflects the small-scale propagation effects, including multi-path effects. Moreover, $\lambda (d_{m}^{k})$ is usually a complex random variable with $E\left[{\left|\lambda \left(d_{m}^{k}\right)\right|}^{2}\right]=1$. The large-scale propagation model in [37,38] is applied as

(4) $$\begin{array}{c} \beta \left(d_{m}^{k}\right)=\left\{\begin{array}{cc} \beta_{0}{\left(d_{m}^{k}\right)}^{-\alpha }, & \mathrm{LoS}\;\mathrm{link},\\ \kappa \beta_{0}{\left(d_{m}^{k}\right)}^{-\alpha }, & \mathrm{NLoS}\;\mathrm{link}, \end{array}\right. \end{array}$$

where $\beta_{0}$ is the path loss at the reference distance $d_{0}=1$ m, α is the path loss exponent, and κ<1 is the additional fading factor when the NLoS link is established. Therefore, considering the randomness of LoS and NLoS link occurrences and the stochastic small-scale propagation effects, the average channel power gain is denoted

(5) $$\begin{array}{cc} E\left[{\left|h\left(d_{m}^{k}\right)\right|}^{2}\right]\; & =P_{LoS}\left(d_{m}^{k}\right)\beta_{0}{\left(d_{m}^{k}\right)}^{-\alpha }+P_{NLoS}\left(d_{m}^{k}\right)\kappa \beta_{0}{\left(d_{m}^{k}\right)}^{-\alpha }\\ \; & ={\hat{P}}_{LoS}\left(d_{m}^{k}\right)\beta_{0}{\left(d_{m}^{k}\right)}^{-\alpha },\;m\in M,\;k\in K, \end{array}$$

where ${\hat{P}}_{LoS}\left(d_{m}^{k}\right)=P_{LoS}\left(d_{m}^{k}\right)+\left(1-P_{LoS}\left(d_{m}^{k}\right)\right)\kappa$ represents the regularized LoS link occurrence probability. Using the homogeneous approximation method described in [37], we can set ${\hat{P}}_{LoS}\left(d_{m}^{k}\right)\approx {\bar{P}}_{LoS}\left(d_{m}^{k}\right)$, where ${\bar{P}}_{LoS}\left(d_{m}^{k}\right)$ is taken as the probability value corresponding to the most likely elevation angle.

Furthermore, when the UAV hovers at CP *k* to collect the data of SN *m*, the data rate is denoted $R\left(d_{m}^{k}\right)=B\log_{2}\left(1+\frac{P_{u}{\left|h\left(d_{m}^{k}\right)\right|}^{2}}{\sigma^{2}\Gamma }\right)$, where *B* represents the channel bandwidth, $P_{u}$ denotes the transmit power of the UAV, $\sigma^{2}$ is the noise power at the BS, and Γ refers to the gap between the SNR required between the actual system and the theoretical model to achieve a specified performance, usually with Γ>1. Then we can obtain an approximation of the mean data transmission rate from Jensen’s inequality, expressed as $E\left[R(d_{m}^{k})\right]\approx B\log_{2}\left(1+\frac{P_{u}E\left[{\left|h\left(d_{m}^{k}\right)\right|}^{2}\right]}{\sigma^{2}\Gamma }\right).$ After that, it is assumed that the sensed data in SN *m* are compressed and packaged into data packets of defined size $L_{m}$. According to Equation (5) and the related description in this section, the data collection time required for SN *m* at CP *k* can be given by

(6) $$\begin{array}{c} \tau_{m}^{k}=\frac{L_{m}}{E\left[R(d_{m}^{k})\right]}=L_{m}/\left(B\log_{2}\left(1+\frac{P_{u}{\bar{P}}_{LoS}\left(d_{m}^{k}\right)\beta_{0}{\left(d_{m}^{k}\right)}^{-\alpha }}{\sigma^{2}\Gamma }\right)\right),\;m\in M,\;k\in K, \end{array}$$

where $E\left[R(d_{m}^{k})\right]$ represents the mean data transmission rate.

The computational model needs to be discussed in two cases. When the energy is sufficient, since the UAV is equipped with an edge server, it acts as both the mobile data collector and the MEC server to further ensure information freshness in computationally intensive scenarios. Specifically, during the flight, the UAV can complete part of the computation task based on the collected data, the percentage of which is denoted $P_{com}$. Then the remaining part is finally offloaded to the MEC server $s_{0}$ for processing. When the energy is limited, to ensure that the data of all SNs are collected and transmitted, i.e., to complete the data collection task while reducing energy consumption, the UAV does not perform the computation task during the flight. Rather, the UAV offloads all data to the MEC server $s_{0}$ until the end of the flight.

### 2.3. AoI Model

At CP *k*, it is assumed that the UAV uses the TDMA scheme for data collection of $\left|A_{k}\right|$ SNs. Let $\Delta_{m}^{k}\left(t\right)$ denote the instantaneous AoI of SN *m* at time t (≥0). Assuming that SN *m* is associated with CP *k*, its AoI can be expressed as

(7) $$\begin{array}{c} \Delta_{m}^{k}\left(t\right)=\max\left\{0,t-t_{m}^{k}\right\},\;m\in M,\;k\in K, \end{array}$$

where $t_{m}^{k}$ denotes the instant when the data of SN *m* are generated. Note that the UAV completes only one round of data collection task, so the data at each SN are updated once during the period *T* in which the UAV performs the task. As a result, for any SN *m*, we are only interested in the AoI between its data generation and its reception by the monitoring center, i.e., $t_{m}^{k}\leq t\leq T$. Moreover, the communication link establishment time and the compression and sampling time are small enough to be ignored. Therefore, denoting the instant by $T_{u}^{k}$ when the UAV reaches CP *k*, it is also equal to the data generation time of the first SN at CP *k* whose data are collected, k∈K.

**Figure 3:**
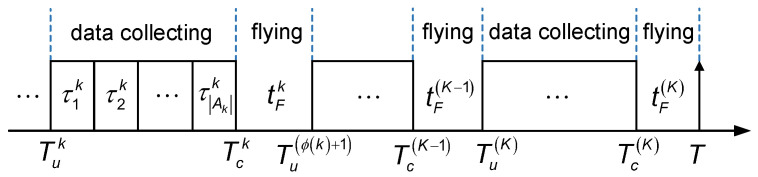
The timeline of the UAV accomplishing the data collection task.

For illustrative purposes, let $q=\left[(1),\cdots ,(k),\cdots ,(K)\right]$ denote the vector of the UAV’s visiting sequences, where (k) gives the subscript of the *k*-th CP in the sequence and (k)∈K. Moreover, assuming that *k* is the ϕ(k)-th CP visited by the UAV, then ϕ(k)∈K and $k=\left(\phi (k)\right)$. As shown in Figure 3, the interval between $T_{u}^{k}$ and $T_{u}^{\left(\phi (k)+1\right)}$ can be divided into two segments, where $T_{c}^{k}$ denotes the instant when the UAV completes the collection of data from all the associated SNs at CP *k*.

The data collection time of the UAV can be derived from (6) as

(8) $$\begin{array}{c} t_{c}^{k}=\sum_{m=1}^{M}o_{m}^{k}\tau_{m}^{k}=\sum_{m=1}^{M}o_{m}^{k}\left[\frac{L_{m}}{B\log_{2}\left(1+\frac{P_{u}{\bar{P}}_{LoS}\left(d_{m}^{k}\right)\beta_{0}{\left(d_{m}^{k}\right)}^{-\alpha }}{\sigma^{2}\Gamma }\right)}\right],\;k\in K,\;m\in M, \end{array}$$

where $o_{m}^{k}$ and $\tau_{m}^{k}$, respectively, represent the association relationship and data collection time required for SN *m* at CP *k*.

The flight time of the UAV can be expressed as

(9) $$\begin{array}{c} t_{F}^{k}=\frac{D_{c}^{k}}{V}=\frac{∥u_{\left(\phi (k)+1\right)}-u_{k}∥}{V},\;k\in K, \end{array}$$

where $D_{c}^{k}$ represents the distance between CP *k* and CP $\left(\phi (k)+1\right)$. In particular, since the UAV eventually returns to the initial position $w_{0}$, it can be obtained that $u_{\left(K+1\right)}=w_{0}$.

Notice that the data at each SN are continuously getting older since they are generated. During the period *T*, the peak AoI of all SNs can be denoted

(10) $$\begin{array}{c} AoI_{p}=\Delta^{\left(1\right)}\left(T\right)=T-T_{u}^{\left(1\right)}=\sum_{k=1}^{K}\left(t_{c}^{k}+t_{F}^{k}\right),\;k\in K, \end{array}$$

where $\Delta^{\left(1\right)}\left(t\right)$ represents the AoI of the first SN whose data are collected at CP (1).

## 3. Problem Formulation

In this section, we present a trajectory planning problem in a UAV-assisted information freshness-oriented MEC network. The peak AoI is minimized by jointly optimizing the locations of CPs, SN-CP associations, as well as the flight trajectory of the UAV. Specifically, the trajectory of the UAV consists of the hover points for data collection $L=\{u_{k}|k\in K\}$ and the visiting sequence q. Let F represent a set of K! (the factorial of *K*) permutations for the visiting sequences of *K* CPs. Then we have

(11) $$\begin{array}{c} q=\left[(1),\cdots ,(k),\cdots ,(K)\right]\in F. \end{array}$$

Analyzing (10), it is clear that $AoI_{p}$ is strongly correlated with *K*. Furthermore, (8) shows that $t_{c}^{k}$ is only related to the SN-CP association relationship o and the distance between CP *k* and the associated SNs. In addition, from (9), it can be found that $AoI_{p}$ is closely related to the trajectory of the UAV. Therefore, we formulate the optimization problem as

(12) $$\begin{array}{cc} (P0):\; & \min_{K,L,q,o}\;\sum_{k=1}^{K}\left(t_{c}^{k}+t_{F}^{k}\right)\\ \; & \begin{array}{cc} s.t.\; & (1)–(3),\;\mathrm{and}\;(11). \end{array} \end{array}$$

Denote the optimal solution of (P0) as $\left(K^{*},L^{*},q^{*},o^{*}\right)$, corresponding to the optimal strategy. Note that the problem (P0) is a very difficult mixed-integer non-convex optimization problem to solve because the variables L and q have dimensions that vary with the integer variable *K*; (2), (3), and (11) are non-convex discrete constraints, and the variables L, q, and o are coupled.

## 4. Proposed Algorithm

In this section, we elaborate on how to solve this optimization problem accurately and efficiently. Since the objective function in (12) is a weighted sum of the data collection time and the UAV’s flight time, (P0) can be decomposed into two sub-problems: the CP location and SN-CP association problem and the UAV trajectory planning problem. Then these two sub-problems are discussed separately to give feasible algorithms. Finally, an iterative two-step algorithm called the AoI-minimized association and trajectory planning (AoI-MATP) algorithm is designed to find the optimal solution to the proposed problem (P0).

### 4.1. CP Location and SN-CP Association Optimization

Firstly, the SN-CP association problem can be described as

(13) $$\begin{array}{cc} (P1):\; & \min_{K,L,o}\;\sum_{k=1}^{K}\sum_{m=1}^{M}o_{m}^{k}\left[\frac{L_{m}}{B\log_{2}\left(1+\frac{P_{u}{\bar{P}}_{LoS}\left(d_{m}^{k}\right)\beta_{0}{\left(d_{m}^{k}\right)}^{-\alpha }}{\sigma^{2}\Gamma }\right)}\right]\\ \; & \begin{array}{cc} s.t.\; & (1)–(3). \end{array} \end{array}$$

According to (13), since the objective function and constraint (1) are both nonlinear, the problem (P1) is a nonlinear programming problem. Furthermore, due to the binary indicator $o_{m}^{k}$, both (2) and (3) are discrete constraints, so Problem (P1) is a very challenging mixed-integer non-convex optimization problem for which there is no generalized algorithm to find an optimal solution. To decrease the complexity of solving this problem, we first choose a suitable clustering algorithm to find the optimal value of *K* and the corresponding SN-CP association. In this context, the binary indicator $o_{m}^{k}=1$ indicates that the data of SN *m* are collected by the UAV at the CP directly above SN *k* and $\tau_{m}^{k}$ denotes the required data collection time; otherwise, $o_{m}^{k}=0$. In addition, constraint (2) ensures that each SN can only be associated with one SN in its neighborhood, where the set $N_{m}$ is used to denote the neighboring nodes of the SN *m*, defined as $N_{m}=\left\{k|∥w_{m}-w_{k}∥\leq r_{u},\;k\in M\right\}$. Therefore, the problem to be solved transforms into

(14) $$\begin{array}{cc} (P2):\; & \min_{o}\;\sum_{k=1}^{M}\sum_{m=1}^{M}o_{m}^{k}\tau_{m}^{k}\\ \; & \begin{array}{cc} s.t.\; & \sum_{k\in N(m)}o_{m}^{k}=1,\forall m,\\ \; & \sum_{k=1}^{M}\max_{m\in N(k)}o_{m}^{k}=K,\\ \; & o_{m}^{k}\in \left\{0,1\right\},\forall m,k. \end{array} \end{array}$$

For a given *K*, it is easy to realize that (P2) is an integer linear programming (ILP) problem that is NP-hard. We propose an algorithm named AP clustering-based collection time optimization algorithm to obtain a suboptimal solution. The algorithm is insensitive to the initial positions of the data points and considers all SNs as candidate exemplars (i.e., cluster centers). Moreover, its input consists of a similarity matrix, and the similarity of SN *k* as the exemplar of SN *m* is denoted s(m,k), which is given by

(15) $$\begin{array}{c} s\left(m,k\right)=\left\{\begin{array}{cc} -\tau_{m}^{k}, & m\neq k,\;m,k\in M,\\ -\varepsilon , & m=k,\;m,k\in M, \end{array}\right. \end{array}$$

where ε denotes a positive clustering parameter that determines the size of *K*.

The algorithm contains two main iterative messages: (1) Responsibility r(m,k) is passed from SN *m* to the candidate exemplar SN *k*, reflecting the degree to which SN *k* is suitable to serve as the exemplar of SN *m*. (2) Availability a(m,k) is passed from the candidate exemplar SN *k* to SN *m*, which reflects the suitability of SN *m* to select SN *k* as its exemplar, taking into account the support of other SNs for SN *k* as the exemplar. The AP algorithm iteratively updates the above two messages as

(16) $$\begin{array}{c} r_{t+1}\left(m,k\right)=s\left(m,k\right)-\max_{k^{\prime }\in N\left(m\right)\setminus \left\{k\right\}}\left\{a_{t}\left(m,k^{\prime }\right)+s\left(m,k^{\prime }\right)\right\}, \end{array}$$

(17) $$\begin{array}{c} a_{t+1}\left(m,k\right)=\left\{\begin{array}{cc} \min\left\{0,r_{t}\left(k,k\right)+\sum_{m^{\prime }\in N\left(k\right)\setminus \left\{m,k\right\}}\max\left\{0,r_{t}\left(m^{\prime },k\right)\right\}\right\}, & m\neq k,\\ \sum_{m^{\prime }\in N\left(k\right)\setminus \left\{m,k\right\}}\max\left\{0,r_{t}\left(m^{\prime },k\right)\right\}, & m=k, \end{array}\right. \end{array}$$

where $r_{t}\left(\cdot \right)$ and $a_{t}\left(\cdot \right)$, respectively, represent the responsibility and availability in the *t*-th iteration, $k^{\prime }$ represents one of the neighboring nodes of SN *m* other than *k*, and $m^{\prime }$ represents one of the neighboring nodes of SN *k* other than *m* and *k*.

The algorithm converges until the sum of similarities of all the data points to their nearest class-representative points is maximized. At that moment, a stable set of exemplars can be obtained, whose indexes are denoted by

(18) $$\begin{array}{c} C=\{k|r(k,k)+a(k,k)>0\}, \end{array}$$

so the SNs are divided into K=|C| clusters. For any m∈M, the index of its exemplar can be given by k=argmaxs(m,k),k∈C, whereby all SN-CP association variables o are easily obtained. Based on this, to find the specific locations of the *K* CPs, for each cluster, the problem of determining the CP location is modeled as a single-facility siting problem and resolved by a simple iterative process of the center of gravity method:

(19) $$\begin{array}{c} u_{k}=argmin\sum_{m=1}^{M}o_{m}^{k}\tau_{m}^{k},\;k\in C. \end{array}$$

The complete procedures are shown in Algorithm 1. Since the algorithm runs for at most $t_{max}$ rounds before convergence, assuming that the computation of (16) and (17) is performed sequentially in each round, the worst-case time complexity is $O(M^{3}t_{max})$, which is polynomial complexity about *M*. In addition, the space complexity of the algorithm is $O(M^{2}t_{max})$, since the storage space for the variables can be reused in each round.
**Algorithm 1** AP-based CP location and SN-CP association optimization algorithm1:Initialize $w_{m}$, ε, $\tau_{m}^{k}$, $t_{max}$, r(m,k)=a(m,k)=0, and t=0.2:Calculate $\tau_{m}^{k}$ between any two SNs, and set s(m,k) according to (15).3:**repeat**4:   Update r(m,k),a(m,k) according to (16) and (17), respectively.5:   Update t←t+1.6:**until** Convergence or $t\geq t_{max}$.7:Find C by (18) and establish *K* and o.8:Find L by (19) according to the center of gravity method.

**Remark** **1.** *For the proposed Algorithm 1, the advantages are as follows. First, it does not need to specify the number of final clusters and different clustering results can be obtained by adjusting the preference −ε. Moreover, the algorithm is insensitive to the initial value of the data and the obtained clustering results are more stable. Furthermore, compared to other clustering algorithms, such as k-centers clustering, the squared error of its results is smaller.*

### 4.2. UAV Trajectory Optimization

Based on the cluster center locations obtained from Algorithm 1, the trajectory planning problem for a UAV is formulated as

(20) $$\begin{array}{cc} (P3):\; & \min_{q}\;\sum_{k=1}^{K}\frac{∥u_{\left(\phi (k)+1\right)}-u_{k}∥}{V}\\ \; & \begin{array}{cc} s.t.\; & (11). \end{array} \end{array}$$

Noting that the objective function and constraints of (P3) are discrete and nonlinear, it is a non-convex optimization problem hard to find a globally optimal solution. Fortunately, it is not difficult to find that it belongs to the combinatorial optimization problem, particularly a variation of the traveling salesman problem (TSP). Thus, there is no generalized polynomial time solution for (P3). To avoid the surge in algorithmic complexity due to the excessive problem size, we propose an elite genetic algorithm (eGA)-based algorithm to find a suboptimal solution to this problem.

The algorithm uses a sequence coding approach, where the visiting sequence of a UAV q is viewed as a chromosome, and each value in q represents a different gene. The objective function in (P3) can be used to construct the fitness function of the algorithm

(21) $$\begin{array}{c} f\left(q\right)=\frac{1}{\sum_{k=1}^{K}\frac{∥u_{\left(\phi (k)+1\right)}-u_{k}∥}{V}}=\frac{V}{\sum_{k=1}^{K}∥u_{\left(\phi (k)+1\right)}-u_{k}∥},\;q\in F. \end{array}$$

The function in (21) is used to evaluate the degree of excellence of an individual: the higher the fitness, the higher the probability that it contributes to finding the optimal solution. Following the principle of survival of the fittest in evolutionary theory, we use the elitist-preserving strategy to select the resulting breeding offspring. More specifically, the most adaptable individual in the current population is retained as an elitist, and a binary tournament selection operator is used for mating selection. After performing the ordered crossover and shuffle indexes mutation operations, if the previously retained elitist of the parent is better than the most adapted individual in the current population, it will be used to replace the worst individual in the current population. To improve the algorithm’s ability to search for local optima, the 2-OPT algorithm is used to optimize the elites in the current population. Then $N_{p}$ optimal individuals in the current population are selected for the next round of evolution. The specific steps are described in Algorithm 2.
**Algorithm 2** eGA-based UAV trajectory optimization algorithm   1:Initialize flight time $t_{F}$ with L, eGA-related parameters $N_{p}$, $N_{g}$, $P_{c}$, $P_{m}$, $P_{opt}$.   2:Generate the initial population with $N_{p}$ chromosomes.   3:Set the index of generation n=0.   4:**repeat**   5:   Update the fitness values of $N_{p}$ chromosomes according to (21) and select the best chromosome remaining.   6:   Select the parent chromosomes with the binary tournament operator for mating.   7:   Perform the ordered crossover operations with probability $P_{c}$ and shuffle indexes mutation operations with probability $P_{m}$ on parent chromosomes.   8:   Perform the 2-OPT algorithm for local optimization with probability $P_{opt}$.   9:   Update the fitness values of new chromosomes and generate the new population with $N_{p}$ chromosomes according to the elitist-preserving strategy. 10:   Update n←n+1. 11:**until** $n\geq N_{g}$. 12:Select the best chromosome in the final generation as the best visiting sequence, i.e., q=argmaxf(q).

Analyzing Algorithm 2, its time complexity mainly depends on lines 7–8, which is approximately $O(N_{g}N_{p}K)$ at worst, and the space complexity is $O(N_{p}K)$.

### 4.3. AoI-MATP Algorithm

Based on Algorithms 1 and 2, we propose an AoI-MATP algorithm to find the optimal solution to the problem (**P0**) approximately.

The algorithm is an iterative process, where each round corresponds to a different ε, leading to varying *K*. The optimal collection time and the corresponding optimal flight time can be obtained by executing Algorithms 1 and 2 alternately. When the algorithm converges, the optimal solution $\left(K^{*},L^{*},q^{*},o^{*}\right)$ can be determined based on the ε corresponding to the minimum $AoI_{p}$. The detailed procedures of the algorithm are described in Algorithm 3. It is well-understood that the complexity of Algorithm 3 is determined by Algorithms 1 and 2, which it contains.
**Algorithm 3** AoI-MATP algorithm   1:Initialize $w_{m}$, a small positive constant Δε, and $\varepsilon =\varepsilon_{0}$.   2:Calculate collection time $\tau_{m}^{k}$ between any two SNs.   3:**repeat**   4:   Run Algorithm 1 to obtain *K*, SN-CP association o, and locations of CPs L with ε.   5:   Calculate the optimal collection time $t_{c}^{k}$ in each CP *k* by (8) and flight time $t_{F}$ between any two CPs according to their distances.   6:   Run Algorithm 2 to obtain the visiting sequence q.   7:   Calculate the optimal peak AoI by (9) and (10).   8:   Update ε←ε−Δε.   9:**until** ε<0. 10:Find the minimum $AoI_{p}^{*}$ with the optimal $\varepsilon^{*}$, and the according optimal solutions $\left(K^{*},L^{*},q^{*},o^{*}\right)$ are all found.

## 5. Simulation Results and Discussion

To evaluate the performance of the proposed algorithm, we present and analyze the simulation results in this section. The simulations were executed on a desktop computer equipped with an Intel Core i7-12700 CPU@2.10 GHz, based on Python 3.8. The manufacturer of the device is HP, sourced from Beijing, China. We consider *M* SNs randomly distributed in a square area with a side length of 2000 m, and the departure time of the UAV from the MEC server $s_{0}$ is t=0. If not specified, the system parameters are set as follows: $H_{u}=50$ m, V=20 m/s, $r_{u}=1000$ m, (a,b)=(9.61,0.16), $\beta_{0}=-60$ dB, α=2.2, κ=0.2, B=1 MHz, $P_{u}=20$ dBm, $\sigma^{2}=-110$ dBm, Γ=8.2 dB.

**Figure 4:**
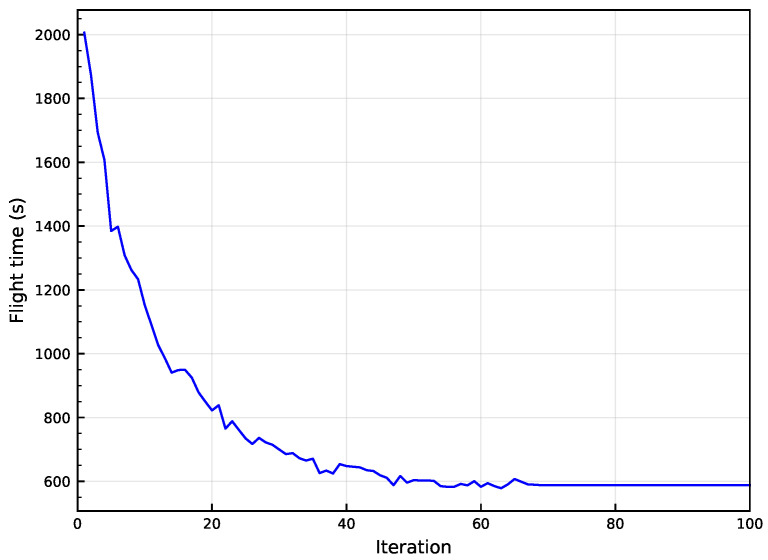
The convergence curve of elite genetic algorithm (eGA) algorithm when K=50.

**Figure 5:**
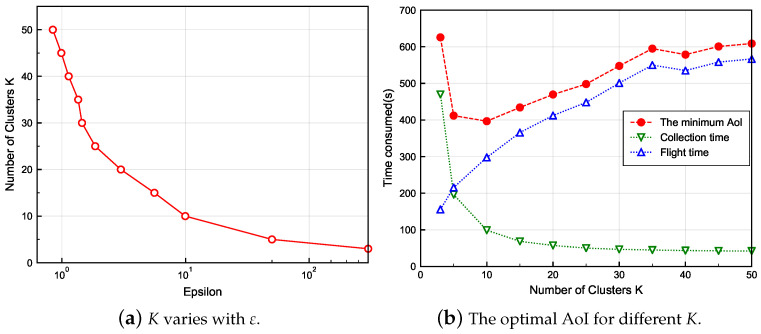
Clustering results and corresponding optimal age of information (AoI) obtained by Algorithm 3 when M=50.

Running our proposed Algorithm 3, the convergence curve of the GA algorithm is shown in Figure 4 when *K* reaches a maximum value of 50. It can be seen that the flight time shows an overall significant decrease with the number of iterations. Convergence is reached when the algorithm has been iterated about 68 times. Figure 5a shows how the number of CPs *K* changes regarding the parameter ε for M = 50. Experimentally, it is found that different ε may lead to the same *K*. To demonstrate the results more clearly, we choose the ε that makes the peak AoI the smallest for each *K*. It is obvious from Figure 5a that *K* tends to decrease as ε increases. According to (15), this is due to the fact that −ε represents the likelihood of each SN to be an exemplar, and the larger −ε is, the more SNs are likely to be exemplars. Similarly, the larger ε is, the fewer SNs are likely to be exemplars, i.e., the smaller *K* is.

Figure 5b demonstrates the optimal peak AoI and the relative collection and flight times for different *K*. The specific simulation results when M=50 are listed in Table 1. In particular, the parameters in Algorithm 2 are set as $N_{p}$ = 300, $N_{g}$ = 300, $P_{c}$ = 0.99, $P_{m}$ = 0.15, $P_{opt}$ = 0.1. First, it can be found from both subfigures of Figure 5 that *K* does not start at 1 but at 3. The reason is that the clustering result with K<3 cannot satisfy the distance limit in (1). Second, it can be observed that the collection time decreases with increasing *K*, while the flight time increases with it. In addition, the collection time decreases fast and dominates at the beginning, but the rate of decrease becomes flat in the latter half, while the flight time changes more uniformly, which leads to a trend of decreasing and then increasing peak AoI. Analyzing the reason, when *K* is larger, there are fewer SNs associated at each CP, the distances between SNs and CPs are shorter, and the collection time decreases consequently. In contrast, the UAV’s flight trajectory contains more CPs, increasing its flight distance and yielding a longer flight time. In particular, it is noted that the peak AoI for the K=50 case reaches 608.76 s. This case implies that the data of each SN are collected directly above itself, and the UAV needs to fly over each SN one by one for data collection. Therefore, the poorer peak AoI is due to the longer flight time caused by the fact that clustering was not performed. From the above analysis, it is easy to conclude that our proposed clustering idea can well strike a balance between the collection time and the flight time, so the optimal peak AoI can be achieved by choosing a suitable *K*.

**Figure 6:**
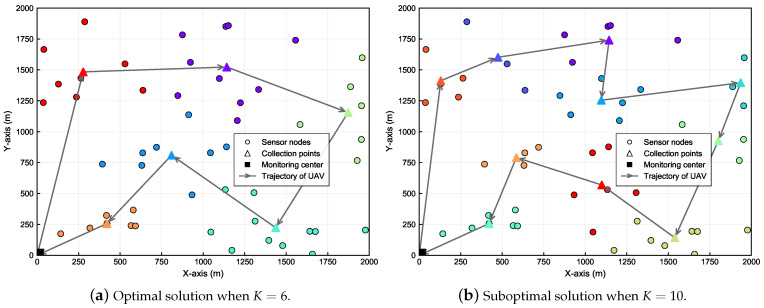
Optimal and suboptimal clustering results and trajectories when M=50.

The optimal and suboptimal clustering results and the flight trajectories are respectively visualized in Figure 6a,b. Note that the different colored circles in the figure represent SNs in different clusters. The specific simulation results are as follows. When K=6, the optimal peak AoI is 393.20 s, corresponding to a collection time of 156.27 s and a flight time of 236.93 s. When K=10, the suboptimal peak AoI is 396.77 s, corresponding to a collection time of 98.98 s and a flight time of 297.79 s.

Further, to better evaluate the performance of the proposed Algorithm 3, the following four schemes are considered as comparisons:

1. Solve the ILP problem (P2) using the branch-and-cut (BC) method and find the optimal trajectory using Algorithm 2.
2. Solve the problem (P2) using Algorithm 1 and find the optimal trajectory using the dynamic programming (DP) algorithm.
3. The first step is the same as in Scheme 2, and then solve the TSP to obtain the optimal trajectory.
4. The first step is the same as above, and the optimal trajectory is solved using the greedy algorithm.

**Figure 7:**
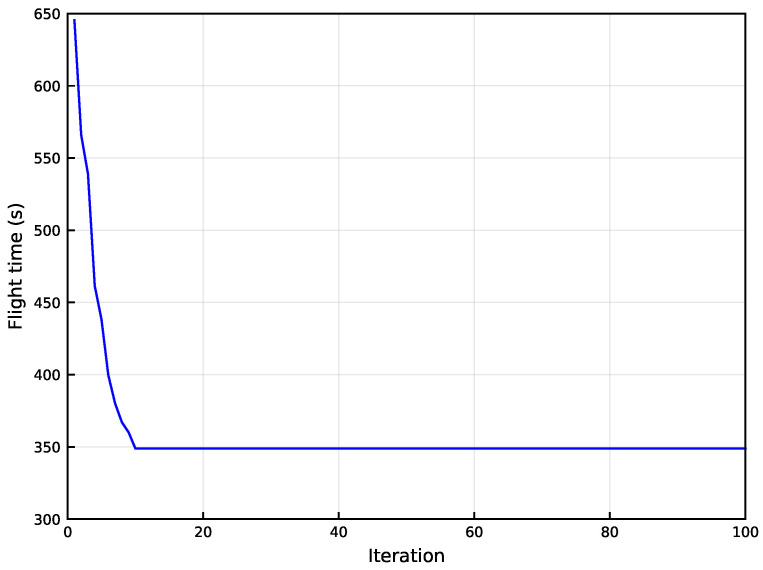
The convergence curve of Algorithm 2 when K=20.

Considering that the complexity of the DP algorithm can be up to $O(2^{M})$, *M* is set to 20 to ensure that the experiment can be carried out properly. Moreover, the parameters in Algorithm 2 are set as $N_{p}$ = 300, $N_{g}$ = 500, $P_{c}$ = 0.9, $P_{m}$ = 0.2, $P_{opt}$ = 0.1. Running Algorithm 3, the convergence curve of the GA algorithm is shown in Figure 7 when *K* reaches a maximum value of 20. It can be seen that the flight time shows an overall significant decrease with the number of iterations. Convergence is reached when the algorithm has been iterated about ten times, less than the number of iterations at K=50. Figure 4 and Figure 7 illustrate that our proposed algorithm can solve the problem efficiently with a small number of CPs and also obtains an asymptotically optimal solution with an acceptable number of iterations when the number of CPs is large.

**Figure 8:**
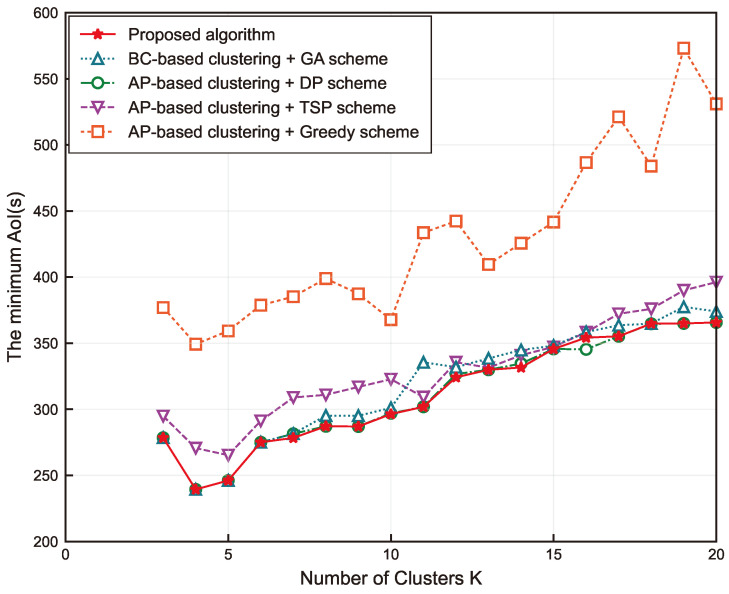
The minimum peak AoI versus *K* when M=20.

The five AoI curves derived from all the schemes are shown in Figure 8 and the specific simulation results are listed in Table 2. It is easily observed that the five curves in the figure have the same trend with *K*. First, there is a small decrease until the optimum is reached, then there is a general upward trend, and all of them reach the worst peak AoI at K=20 except Scheme 4. Slightly different from the AoI curves in Figure 6b, except for Scheme 3 which achieves the optimal peak AoI at K=5, the other three schemes as well as our proposed algorithm all achieve the optimal solution at K=4, and the optimal peak AoI becomes smaller. Understandably, this is because the reduction in *M* leads to the association of fewer SNs at each CP as well. This further results in a lower percentage of data collection time, and the flight time dominates in this case. It can be noticed that Scheme 4 provides the worst peak AoI performance in all cases. This is due to it only considering the nearest unvisited CPs at each decision step and bot being motivated by a global perspective, causing it to obtain usually locally optimal solutions.

On the contrary, Schemes 1 and 2 and the proposed algorithm demonstrate the best performance, and the three curves have identical results for K<9 as well as K>16. Comparing Scheme 1 and the proposed algorithm, since BC is an exact algorithm for solving the ILP problem, the clustering results it obtains must be optimal. However, its peak AoI is not always smaller than the proposed algorithm, such as when K=11 and 14. This is mainly related to the fact that peak AoI includes both collection time and flight time, and the optimal collection time does not necessarily correspond to the optimal flight time. The comparison of Scheme 2 and the proposed algorithm shows that Scheme 2 has better performance at some points with the same clustering results. This is because the DP algorithm included in Scheme 2 obtains the optimal solution by comparing all the candidate paths. Therefore, DP has an exponential algorithm complexity that increases rapidly as the problem size grows. The space required by the algorithm exceeds the memory capacity of the computer when M>20. However, GA belongs to heuristic algorithms; it usually obtains the optimal solution or suboptimal solution but has a better computational complexity than DP. The proposed algorithm can achieve a performance extremely close to the exact algorithm near the optimal number of clusters or when *K* is very large. Overall, Scheme 2 does not apply to the large-scale SNs’ data collection problem, while our proposed algorithm is still able to solve with high accuracy by adjusting the parameters.

Lastly, the performance of Scheme 3 is substantially better than that of Scheme 4, but it still has some gaps with the optimal performance. This can be explained because the TSP always considers the optimal solution of a loop that traverses all CPs from the starting point and back to the starting point. Even if we have subtracted the distance from the starting point to the first visited CP, the result obtained is not the path that makes the peak AoI optimal.

As presented in Table 3, we also evaluated the running time of the proposed algorithm. Combined with the results in Figure 8, it is easy to notice that Schemes 1, 3, and 4 obtain relatively poor solutions in a faster time. The remaining two better-performing schemes vary greatly in their running time. Consistent with our theoretical analysis, Scheme 2 obtains the optimal result with the longest running time. When M=20, the proposed algorithm obtains very similar results to Scheme 2 in a much shorter time. When M>20, the computational complexity of Scheme 2, which contains the DP algorithm, is too high for the simulation to proceed. We give the running times of the remaining schemes for M=30. The four schemes spend more time on computations when *M* is increased, but it is still acceptable.

To better explain the generality of our algorithm, the main simulation results of the four schemes for M=30 are presented in Table 4. The minimum AoI for all schemes is obtained at K=5, and the first three schemes have the same result of 336.25. Only Scheme 4 has a worse result of 413.47. From the simulation results, we can conclude that our algorithm can still give a better feasible solution in an acceptable time at M=30. In agreement with our theoretical analysis of the complexity of the GA algorithm, this is because *K* is very small compared to $N_{p}$ and $N_{g}$, so our algorithm is equally applicable to larger problem sizes. Asymptotically optimal solutions can be obtained by adjusting the size of the hyperparameters in the proposed algorithm if high-precision results are required and computational resources are sufficient.

## 6. Conclusions

In this paper, we mainly consider a UAV-assisted MEC network in which the peak AoI of the collected data is minimized by jointly optimizing the SN-CP associations, location of the data CPs, and UAV trajectory. To solve this complex mixed-integer non-convex optimization problem, we propose an iterative algorithm to first obtain the optimal SN-CP associations and locations of CPs, based on which the optimal UAV trajectory is found. Simulation results show that our proposed algorithm can achieve an excellent balance between the data collection time and the flight time of the UAV and has a superior performance close to several exact algorithms. In future work, we will consider the case where the UAV acts as the MEC server in computation-intensive tasks, e.g., by optimizing the allocation of communication resources to reduce the AoI and energy consumption.

## Figures and Tables

**Table 1:** Simulation results when M=50.

Metric	K = 3	K = 10	K = 20	K = 30	K = 40	K = 50
Minimum Peak AoI (s)	625.70	396.77	469.65	547.69	578.53	608.76
Collection Time (s)	469.96	98.98	57.38	46.78	43.51	42.11
Flight Time (s)	155.74	297.79	412.27	500.92	535.02	566.65

**Table 2:** Simulation results when M=20.

Scheme	K = 3	K = 4	K = 5	K = 10	K = 15	K = 20
Proposed Algorithm	278.50	239.38	246.14	296.37	345.66	365.70
Comparison Scheme 1	278.50	239.38	246.14	301.09	348.14	365.70
Comparison Scheme 2	278.50	239.38	246.14	296.37	345.66	365.70
Comparison Scheme 3	294.86	270.64	265.29	322.77	347.22	396.19
Comparison Scheme 4	377.02	349.27	359.28	367.77	441.64	531.04

**Table 3:** The execution time between the compared algorithms.

	Proposed	Scheme 1	Scheme 2	Scheme 3	Scheme 4
M = 20	78.06 s	17.24 s	569.62 s	118.46 s	61.94 s
M = 30	411.15 s	98.96 s	/	354.62 s	158.75 s

**Table 4:** Simulation results when M=30.

Scheme	K = 4	K = 5	K = 6	K = 10	K = 20	K = 30
Proposed Algorithm	356.38	336.25	346.33	387.07	442.97	495.97
Comparison Scheme 1	356.38	336.25	348.48	400.54	466.49	577.23
Comparison Scheme 3	356.38	336.25	346.33	387.98	445.37	683.11
Comparison Scheme 4	436.47	413.47	471.17	534.57	535.50	625.52

## Data Availability

Data are contained within the article.
